# Mammalian Tyrosyl-DNA Phosphodiesterases in the Context of Mitochondrial DNA Repair

**DOI:** 10.3390/ijms20123015

**Published:** 2019-06-20

**Authors:** Shar-yin Naomi Huang, Yves Pommier

**Affiliations:** Developmental Therapeutics Branch and Laboratory of Molecular Pharmacology, Center for Cancer Research, National Cancer Institute, NIH, Bethesda, MD 20892, USA

**Keywords:** mitochondrial DNA, topoisomerases, TDP1, TDP2, ROS, SCAN1, neurological disorders, cancer, antibiotics and antiviral agents

## Abstract

Mammalian mitochondria contain four topoisomerases encoded in the nuclear genome: TOP1MT, TOP2α, TOP2β, and TOP3α. They also contain the two known tyrosyl-DNA phosphodiesterases (TDPs): TDP1 and TDP2, including a specific TDP2^S^ isoform. Both TDP1 and TDP2 excise abortive topoisomerase cleavage complexes (TOPccs), yet their molecular structures and mechanisms are different. TDP1 is present across eukaryotes, from yeasts to humans and belongs to the phospholipase D family. It functions without a metal cofactor and has a broad activity range, as it also serves to cleanse blocking 3′-DNA ends bearing phosphoglycolate, deoxyribose phosphate, nucleoside, nucleoside analogs (zidovudine), abasic moieties, and with a lower efficiency, TOP2ccs. Found in higher vertebrates, TDP2 is absent in yeast where TDP1 appears to perform its functions. TDP2 belongs to the exonuclease/endonuclease/phosphodiesterase family and requires magnesium as a cofactor to excise TOP2ccs, and it also excises TOP1ccs, albeit with a lower efficiency. Here, we review TDP1 and TDP2 in the context of mitochondrial DNA repair and discuss potential new research areas centered on the mitochondrial TDPs.

## 1. Introduction

Nuclear DNA repair pathways are critical for genomic stability and their genetic defects have been linked with cancer predisposition (for instance, mismatch repair deficiencies in colon cancers and BRCA deficiencies in breast and ovarian cancers), neurological diseases (ataxia), and immunological diseases (severe combined immunodeficiency). They are also the targets of recently approved therapeutic agents (such as poly (ADP-ribose) polymerase [PARP] inhibitors) and the focus of intense research. In contrast, repair pathways for the mitochondrial genome remain far less studied and understood. Our current understanding has overturned the early perception that only rudimentary DNA repair pathways exist in the mitochondria. Although damaged mitochondrial DNA (mtDNA) can be extensively degraded and replaced by new intact mtDNA [1,2,3,4], it is not the only repair mechanism. In fact, DNA repair in the mitochondria partially mirrors the intricate network of DNA repair pathways found in the nucleus (see reviews [5,6]).

Here, we focus on the repair of mtDNA in response to topoisomerase-induced DNA damage. Topoisomerases are a family of enzymes essential for many fundamental nucleic acid metabolic processes, including replication, transcription, recombination, and chromosome segregation. Topoisomerases are also specifically targeted by widely used front-line anti-cancer therapeutics (doxorubicin, etoposide, mitoxantrone, topotecan, and irinotecan) and antibiotics (ciprofloxacin and other quinolones). Decades of research efforts have revealed many redundant parallel repair pathways for topoisomerase-induced DNA damage in the nucleus. Interestingly, several recent reports demonstrated that topoisomerases and the repair proteins for trapped topoisomerases collectively function in the maintenance of the mitochondrial genome.

**Figure 1:**
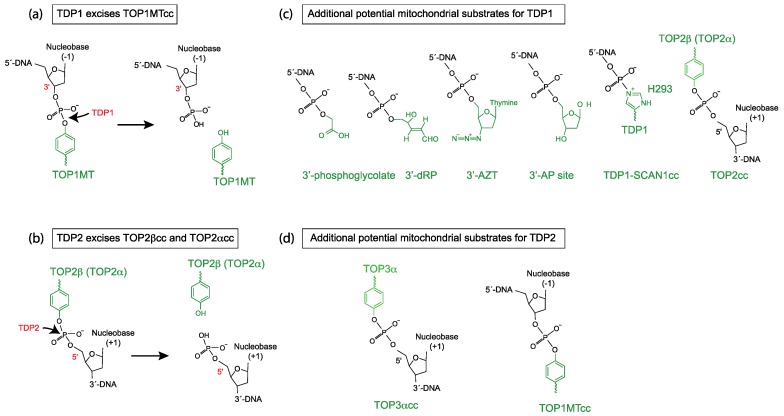
Scheme of biochemical reactions and potential mitochondrial substrates for tyrosyl-DNA phosphodiesterases 1 and 2 (TDP1 and TDP2). (**a**) Biochemical reaction of TDP1 hydrolyzing a 3′-tyrosyl-DNA bond (TOP1MT cleavage complex in this case). (**b**) Biochemical reaction of TDP2 hydrolyzing a 5′-tyrosyl-DNA bond (TOP2 cleavage complex in this case). (**c**) Structures of additional potential mitochondrial substrates for TDP1. Note that TDP1 undergoes an intermediate step where it forms a covalent bond between its H293 residue and the 3′-end of the DNA (TDP1-SCAN1cc). This intermediate is probably stabilized and potentially pathogenic in SCAN1 patients with the mutation H493R (reviewed in [9]). (**d**) Structures of additional potential mitochondrial substrate for TDP2.

Topoisomerases share a common catalytic mechanism where they cycle through a transient intermediate, termed topoisomerase cleavage complex (TOPcc), in which they cleave the DNA by forming a covalent bond between their catalytic tyrosine and one of the DNA ends. The family of topoisomerases is classified into different subtypes depending on whether the topoisomerase cleaves one or both DNA strands and the polarity of covalent linkages. TOP1cc is linked to the DNA via a 3′-tyrosyl-DNA bond while TOP2cc and TOP3cc are linked to the DNA with the opposite polarity, a 5′-tyrosyl-DNA bond (see Figure 1). The covalent bonds between topoisomerases and DNA have to reverse to allow the DNA backbone to be resealed and the topoisomerases to be released for a new catalytic cycle (see review [7]).

Many factors stabilize the TOPcc intermediates, such as nearby DNA damage (mismatches, oxidized bases, nicks, abasic sites) [7], drugs that bind at the enzyme-DNA interface [7,8], specifically blocking the religation of TOP1ccs (topotecan and irinotecan), or TOP2ccs (doxorubicin, etoposide, mitoxantrone). Consequently, a network of repair pathways protect the nuclear genome from the damage generated by TOPccs (see review [7]). Tyrosyl-DNA phosphodiesterase 1 and 2 (TDP1 and TDP2) are the two enzymes known to specifically participate in the respective repair pathways for TOP1ccs and TOP2ccs (see review [9,10]). The two enzymes have complementary preferences in terms of substrate polarities. TDP1 hydrolyzes 3′-tyrosyl-DNA bonds more efficiently, while TDP2 is more efficient at hydrolyzing 5′-tyrosyl-DNA bonds (the biochemical reactions are shown in Figure 1a,b). In addition, TDP1 and TDP2 also process a number of biologically relevant substrates, many are potentially present in the mitochondria (Figure 1c,d and discussed below).

**Figure 2:**
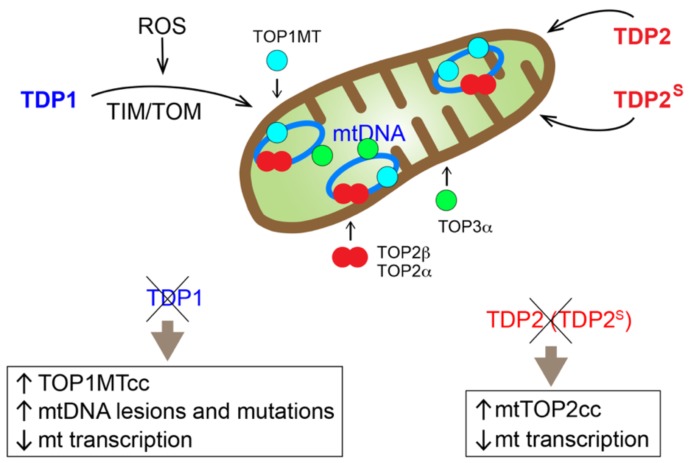
Overview of the mitochondrial topoisomerases and TDPs, all of which are encoded in the nuclear genome and translocate to the mitochondria. TOP1MT, TOP3α and a shorter isoform of TDP2, termed TDP2^S^, contain a canonical cleavable mitochondrial targeting sequence (MTS). TDP1 is transported to mitochondria through the TIM/TOM transport system. Full-length TDP2, TOP2α, and TOP2β do not contain canonical cleavable MTS, and the mechanisms of their mitochondrial translocation have not been defined. The consequences on the mitochondria when mitochondrial TDP1 or TPD2 is eliminated are also listed.

Four topoisomerases are known to function in mitochondria: TOP1MT, TOP2α, TOP2β, and TOP3α [11,12,13] (Figure 2). These enzymes are encoded by the nuclear genome while the mitochondrial genome encodes 13 essential polypeptides for respiratory electron transport chain complexes, as well as the tRNAs and rRNAs necessary for mitochondrial translation. TOP1MT is the only mitochondria-specific topoisomerase due to the presence of a canonical cleavable mitochondrial targeting sequence (MTS) in its N-terminus [12,14]. TOP2α and TOP2β both lack a canonical MTS, and the same TOP2α and TOP2β polypeptides are found in the nucleus and the mitochondria [13]. In contrast, mitochondrial and nuclear forms of TOP3α arise from different translation start sites on the same transcript, where the longer TOP3α polypeptide contains an additional canonical cleavable MTS, specifically directing it to the mitochondria [11] (Figure 2).

## 2. Mitochondrial TDP1 and Its Broad Activity as a 3′-end Cleansing Enzyme

TDP1 was discovered in the late 90s due to its unique enzymatic activity capable of hydrolyzing 3′-tyrosyl-DNA bonds [15,16]. As the only known instances of this type of linkage take place in the catalytic cycle of TOP1, TDP1 was correctly presumed to function in the repair of TOP1cc [17,18]. Conserved across eukaryotes [15,16,17], TDP1 is capable of removing trapped TOP1ccs without eliminating any DNA contents at the DNA damage sites, allowing faithful DNA repair. Additional studies revealed that TDP1 can hydrolyze a broad spectrum of 3′-DNA lesions [19,20,21,22], including 3′-phosphoglycolate (3′-PG) and 3′-deoxyribose phosphate ends (3′-dRP), both common products of oxidative DNA damage (Figure 1c). Although the biochemical activity of TDP1 for the 3′-PG and 3′-dRP is weaker compared to its activity for 3′-phosphotyrosine (mimetic of TOP1cc), genetic analysis showed that TDP1 protects cells from oxidative and alkylation DNA damage [22,23,24,25]. Despite its weak biochemical activity for 5′-phosphotyrosine (mimetic of TOP2cc), genetic studies also suggest that TDP1 acts as a backup repair for TOP2ccs [22,26,27]. These results demonstrate that TDP1 is involved in multiple DNA repair pathways, not just that of TOP1cc repair.

As researchers gradually elucidated the functions of mitochondrial topoisomerases over the years [11,12,13], new methodology and reagents led to the detection of TOP1MTccs and investigation of TOPcc repair in the mitochondria [1,28]. In addition to translocating to the nucleus, TDP1 was found to localize to mitochondria although it does not bear a canonical cleavable MTS [29,30]. A new report demonstrated that reactive oxygen species (ROS) activate the translocation of TDP1 into mitochondria in a TIM/TOM-dependent manner [25] (Figure 2). As TDP1 is an established component of single-strand break repair (SSBR), which is part of base excision repair (BER) in the nucleus [24,31], it is logical for TDP1 to participate in the same pathway in the mitochondria [32]. In confirmation, TDP1 was found to form a complex with ligase III, another key component of SSBR and BER, in the mitochondria [25].

At the DNA level, TDP1 protects mtDNA from accumulating TOP1MTccs [33] and mtDNA lesions/mutations [25]. Because TDP1 also has 3′-nucleosidase activity, several studies have shown that TDP1 is also important in repairing DNA damage induced by anti-viral and anti-cancer nucleoside analogs [34,35,36]. In particular, TDP1 protects mtDNA from damage induced by anti-viral nucleoside analog treatments, such as zidovudine (AZT) [34] (Figure 1c). At the transcription and translation level, lack of TDP1 leads to decreased mitochondrial transcription and reduced OXPHOS function [25,33]. Combined, these experimental data show that TDP1 participates in the repair of trapped TOP1MTcc and oxidative mtDNA damage.

At the mechanistic and biochemical levels, similar to TOP1, TDP1 does not require a metal cofactor or ATP. TDP1 goes through a similar transient intermediate in its catalytic cycle where it is covalently linked to the 3′-end of the DNA (see review [9]). A genetic mutation (H493R) at the active site of TDP1 interferes with the hydrolysis of the TDP1-DNA covalent intermediate and leads to greatly reduced TDP1 catalytic activity, as TDP1 itself becomes trapped on the DNA [37]. The H493R mutation in *TDP1* leads to spinocerebellar ataxia with axonal neuropathy (SCAN1), a rare human disorder affecting non-replicating neuronal cells [38]. Incidentally, the only known enzyme that can resolve the mutant TDP1 covalently-linked to DNA is the wild-type TDP1 [39] (TDP1-SCAN1cc in Figure 1c). It remains to be clarified whether the deficiency to repair stalled topoisomerases or the deficiency to repair oxidative DNA damage or the accumulation of covalently-linked TDP1 constitute the molecular basis of the SCAN1 neurological pathology. Furthermore, it is not established whether the impact of mutant TDP1 on the nuclear or mitochondrial genome is the main contributor to the disease (see review [37]). These intriguing questions will take further efforts to answer. Nevertheless, it is clear that TDP1 is crucial in removing trapped TOP1MTccs and other types of DNA damage on the 3′-ends of mtDNA. This is significant because mtDNA is susceptible to several types of oxidative DNA damage due to the proximity of mtDNA with the mitochondrial oxidative phosphorylation respiratory chain, a major source of ROS.3. Mitochondrial TDP2 and Its Isoforms.

The DNA repair function of TDP2, the counterpart DNA repair enzyme of TDP1, was discovered a decade after TDP1 [40]. Found and conserved in higher vertebrates, TDP2 hydrolyzes 5′-tyrosyl-DNA bonds efficiently and is important for repairing trapped TOP2 after treatment of TOP2 poisons, such as etoposide and doxorubicin [41,42,43]. It is part of the exonuclease/endonuclease/phosphor-diesterase (EEP) family, requires magnesium as a cofactor [40,44], and is structurally and mechanistically different from TDP1 (see review [17]). In humans, inactivation of *TDP2* leads to spinocerebellar ataxia, autosomal recessive 23 (SCAR23), a neurological disease associated with epilepsy, intellectual disability, seizures, and ataxia [45,46].

**Figure 3:**
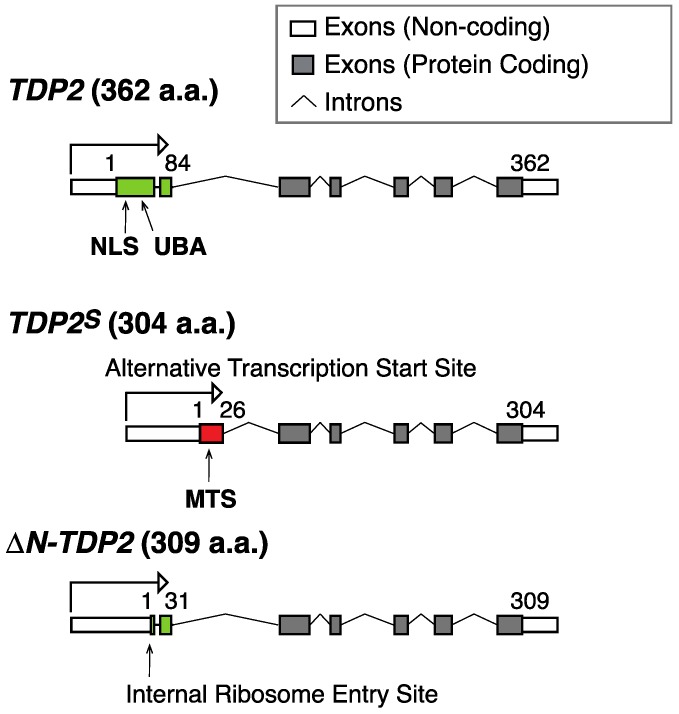
Scheme for the genomic organization of different TDP2 isoforms. The canonical TDP2 isoform encodes a 362 amino acid (a.a.) polypeptide with a nuclear localization sequence (NLS) and a ubiquitin binding domain (UBA) at its N-terminus (green). A shorter isoform termed TDP2^S^ results from an alternative transcription start site, which contains a canonical cleavable mitochondrial targeting sequence (MTS) (red), a unique feature among different isoforms of TDP2 [49]. A recently described ∆N-TDP2 isoform (309 a.a.), which results from a downstream alternative translation start site, lacks NLS, UBA, and MTS [50].

Early studies noted that there were apparently several cellular TDP2 isoforms, and it was unclear whether the different isoforms possessed distinct functions [47]. Although the majority of TDP2 localizes to the nucleus, cytoplasmic expression was clearly visible depending on the cell lines [47,48]. A recent report demonstrated that a shorter isoform of TDP2, termed TDP2^S^, arises from an alternative transcription start site [49] (Figure 3). TDP2 and TDP2^S^ share identical sequences for the catalytic domain except at the N-terminus, where a nuclear-localization sequence is present in canonical TDP2 and a cleavable MTS is found in TDP2^S^ (Figure 3). Consequently, canonical TDP2 preferentially localizes to the nucleus, while TDP2^S^ localizes to the mitochondria and the cytosolic compartments. The same study also found that a small fraction of the canonical TDP2 isoform localizes to mitochondria through undefined transport mechanism. Similar to *TDP1*, deleting *TDP2* or *TDP2^S^* leads to reduced mitochondrial transcript levels and sensitizes cells to TOP2 poisons specifically targeting mitochondria, suggesting that TDP2 is important in repairing trapped TOP2 in the mitochondria [49]. A separate study reported that another short isoform of TDP2, which is equally diffused throughout the cytoplasm and nucleus, is a product of alternative translational start site driven by an internal ribosome entry site [50] (Figure 3). It is possible that both alternative translational and transcriptional start sites contribute to the shorter TDP2 isoforms. On the other hand, the dynamic range of expression of different TDP2 isoforms across different human cancer cell lines could, in fact, be a reflection of distinct regulatory mechanisms in different cell lines.

Notably, a recent case report of a SCAR23 patient [46] with the previously described *TDP2* splicing junction mutation leading to nonsense mRNA decay [45], chronicled phenotypes of mitochondrial dysfunction due to TDP2 deficiency. In particular, the muscle biopsy of the SCAR23 patient displayed profound electron transport chain reduction [46]. It has been proposed that mitochondrial dysfunctions of SCAR23 patients could represent secondary effects resulting from genomic alterations due to lack of nuclear TDP2 [46]. Nevertheless, these clinical findings are also consistent with the importance of TOP2 and TDP2 for the maintenance of the mitochondrial genome. Although there was no detectable mitochondrial dysfunction in the skin fibroblasts obtained from the same SCAR23 patient, the discrepancy can potentially be attributed to the high variability of mitochondrial burden from different tissues [46].

## 3. Perspectives and Open Questions

Our understanding of mitochondrial DNA repair pathways for topoisomerases lags behind that of their nuclear counterparts. While significant advances were made in recent years, many challenging questions remain. In particular, it is not known if TDPs are subjected to post-translational modifications (PTM) or form protein complexes in the mitochondria. It will be crucial to ascertain if TDPs contribute to certain types of mitochondrial diseases, and how they might affect the complex interplay between the nucleus and mitochondria.

While the PTMs of topoisomerases or topoisomerase cleavage complexes in the nucleus are under intense study, it is currently not clear if the topoisomerases in the mitochondria are regulated by PTMs. TDP2 activity has recently been reported to be regulated by phosphorylation by ERK3 [51], and TDP1 is known to be regulated by phosphorylation, ADP-ribosylation, and SUMOylation [52,53,54,55]. Specifically, phosphorylation of TDP1 at the serine 81 position has been shown to be crucial for forming large repair complexes [52], but blocking phosphorylation at this position did not impact the ROS-activated translocation of TDP1 [25]. It remains to be tested whether other PTMs regulates the activities of TDPs in the mitochondria. 

Other than one report showing TDP1 interaction with ligase III in the mitochondria [25], we do not know what other proteins take part in the repair of topoisomerase-induced mtDNA damage. Interrogating the proteins that are known to take part in the repair of nuclear topoisomerase-induced DNA damage will be a reasonable starting point. In the nucleus, both replication and transcription induce trapped TOPccs, which are ubiquitylated and degraded by the proteasome and can be repaired by other endonucleases in TDP-independent pathways (reviewed in [7]). The nature and causes of topoisomerase-induced DNA damage in the mitochondria are less well-defined. Drugs that selectively target mitochondrial topoisomerases remain limited [28,49]. It is also possible to use genetically engineered toxic topoisomerases that accumulate TOP1MTcc in mitochondria [1] to explore the potential roles of TDPs in repairing TOPccs.

Recent reports demonstrated that mitochondria are linked to tumorigenesis via both metabolic and signaling pathways, providing a rationale for targeting mitochondria in anti-cancer therapy (reviewed in [56]). Research focusing on specific delivery of therapeutics to the mitochondria have shown promising results and the efforts in this arena hold great potentials [57,58,59,60,61]. Indeed, TOP1MT has been shown to be critical for tumor development [62]. Although inhibition of mitochondrial TDP1 or TDP2 is expected to have somewhat different effects than inhibiting mitochondrial topoisomerases, the two processes would be intricately linked. It would be worthwhile for future research to distinguish the tumor types that respond to therapies specifically targeting mitochondrial function and to better characterize the molecular background of these tumor types. Another point of consideration is that many therapeutic agents targeting nuclear DNA may also inadvertently target mitochondrial DNA, a fact that is important to bear in mind when interpreting experimental data. For example, some topoisomerase poisons (topotecan, doxorubicin, and lamellarins [28,63]) enter both the nucleus and the mitochondria, and the observed effects can be due to trapped topoisomerases in either or both cellular compartments [63,64]. Another example is antiviral nucleoside analogs that target viral polymerases but not human nuclear polymerases. Incidentally, these antiviral drugs potentially affect mitochondrial polymerases, leading to unintended and unwanted side-effects [34]. Since TDP1 and TDP2 are rational anti-cancer drug targets, it will be beneficial to verify how potential inhibitors will impact TDP1 and TDP2 in the nucleus vs the mitochondria.

TDP1 is capable of removing a ribonucleotide from 3′-ends of DNA, while TDP2 is known to hydrolyze 5′-tyrosyl RNA bonds [20,34,44]. As TOP1 and TOP2 can be trapped by newly incorporated ribonucleotides [44,65], further studies are warranted to determine whether TDPs play any role in the repair of RNA damage. Furthermore, repair of RNA damage by the TDPs can potentially take place in the nuclear, mitochondrial or cytosolic compartments, either induced by or independent of TOPccs. Finally, it remains to be seen whether TDP2 is involved in the repair of TOP3ccs either in the nucleus or mitochondria.

## Abbreviations

CC	Cleavage complex
TDP	Tyrosyl-DNA phosphodiesterase
mtDNA	Mitochondrial DNA
SCAN1	Spinocerebellar ataxia with axonal neuropathy
SCAR23	Spinocerebellar ataxia, autosomal recessive 23
MTS	Mitochondrial targeting sequence
BER	Base excision repair
SSBR	Single-strand break repair
